# Advice to Rest for More Than 2 Days After Mild Traumatic Brain Injury Is Associated With Delayed Return to Productivity: A Case-Control Study

**DOI:** 10.3389/fneur.2019.00362

**Published:** 2019-04-12

**Authors:** Noah D. Silverberg, Thalia Otamendi

**Affiliations:** ^1^Rehabilitation Research Program, GF Strong Rehab Centre, Vancouver, BC, Canada; ^2^Division of Physical Medicine & Rehabilitation, University of British Columbia, Vancouver, BC, Canada; ^3^Sport and Exercise Psychology Laboratory, School of Kinesiology, University of British Columbia, Vancouver, BC, Canada

**Keywords:** brain concussion, craniocerebral trauma, rehabilitation, rest, return to work

## Abstract

**Objectives:** Recent expert agreement statements and evidence-based practice guidelines for mild traumatic brain injury (mTBI) management no longer support advising patients to “rest until asymptomatic,” and instead recommend gradual return to activity after 1–2 days of rest. The present study aimed to: (i) document the current state of de-implementation of prolonged rest advice, (ii) identify patient characteristics associated with receiving this advice, and (iii) examine the relationship between exposure to this advice and clinical outcomes.

**Methods:** In a case-control design, participants were prospectively recruited from two concussion clinics in Canada's public health care system. They completed self-report measures at clinic intake (Rivermead Post-concussion Symptom Questionnaire, Personal Health Questionnaire-9, and Generalized Anxiety Disorder-7) as well as a questionnaire with patient, injury, and recovery characteristics and the question: “Were you advised by at least one health professional to rest for more than 2 days after your injury?”

**Results:** Of the eligible participants (*N* = 146), 82.9% reported being advised to rest for more than 2 days (exposure group). This advice was not associated with patient characteristics, including gender (95% CI odds ratio = 0.48–2.91), race (0.87–6.28) age (0.93–1.01), a history of prior mTBI(s) (0.21–1.20), or psychiatric problems (0.40–2.30), loss of consciousness (0.23–2.10), or access to financial compensation (0.50–2.92). In generalized linear modeling, exposure to prolonged rest advice predicted return to productivity status at intake (B = −1.06, chi-squared(1) = 5.28, *p* = 0.02; 64.5% in the exposure group vs. 40.0% in the control were on leave from work/school at the time of clinic intake, 19.8 vs. 24% had partially returned, and 11.6 vs. 24% had fully returned to work/school). The exposure group had marginally (non-significantly) higher post-concussion, depression, and anxiety symptoms.

**Conclusions:** mTBI patients continue to be told to rest for longer than expert recommendations and practice guidelines. This study supports growing evidence that prolonged rest after mTBI is generally unhelpful, as patients in the exposure group were less likely to have resumed work/school at 1–2 months post-injury. We could not identify patient characteristics associated with getting prolonged rest advice. Further exploration of who gets told to rest and who delivers the advice could inform strategic de-implementation of this clinical practice.

## Introduction

In the early twenty-first century, complete rest until symptom resolution was introduced as best practice for mild traumatic brain injury (mTBI) management (1, 2). This practice was widely disseminated (3, 4) and followed (5, 6). Despite a lack of evidence for efficacy or agreement about what constitutes “rest,” the majority of observational studies (7–10) and randomized clinical trials (11–13) have found that rest beyond few days after mTBI is not beneficial, and may actually prolong recovery (14). Concerns have also been raised about iatrogenic physiological and psychological effects of prescribed rest. For example, removing individuals from their recreational, occupational, and social settings may increase their risk of developing depression (15–17). Correspondingly, the most recent expert consensus statements and practice guidelines for both sport-related mTBI and mTBI sustained in other settings advise against complete rest for more 1 or 2 days (18–21). In Canada, the setting of the present study, this change came in 2013, with the 2nd edition of the Guidelines for Concussion/Mild Traumatic Brain Injury & Persistent Symptoms (22). However, as with changes to practice guidelines in other health conditions, de-implementation of prescribed rest for mTBI may be slow (23, 24).

The present study had multiple objectives. First, we assessed the degree to which patients with mTBI are still being advised to rest for longer than contemporary guidelines recommend. We hypothesized that most participants would still receive prolonged rest advice, as knowledge regarding mTBI management has evolved rapidly and active knowledge-transfer has likely been insufficient (25). Second, we explored whether certain patient characteristics were associated with receiving guideline non-compliant prolonged rest advice. We hypothesized that demographic (e.g., gender), health history (e.g., prior mTBIs), and injury variables (e.g., presence of loss of consciousness) would make it more likely for clinicians to recommend excessive rest. Knowing which patients are more or less likely to be told to rest can inform de-implementation strategies. Third, we aimed to add to the growing literature on the benefits and harms of excessive rest by comparing patients who were vs. were not exposed to prolonged rest advice. We hypothesized that prolonged rest advice would not be strongly related to symptomatic recovery (11, 13), but would coincide with taking longer to return to work/school and having more depressive or anxiety symptoms. These potential harms of prescribed rest have been previously hypothesized (15, 26) but not empirically evaluated.

## Materials and Methods

### Participants and Procedures

This study was a secondary analysis of a broader clinical trial, which examines the effect of tailored follow-up letters on proactive mTBI symptom management by family physicians (ClinicalTrials.gov Identifier: NCT03221218). The study was approved by the University of British Columbia Clinical Research Ethics Board and Fraser Health Research Ethics Board. Participant recruitment took place from August 2017 to October 2018 in two public sector outpatient mTBI clinics in urban British Columbia, Canada.

At their first clinic appointment, patients were screened for eligibility with the following study criteria: (1) aged 18–60 years; (2) sustained a physician diagnosed MTBI < 3 months ago; (3) fluent in English; and (4) had a family physician or could identify a walk-in clinic where they access primary care (for the parent study). After consenting, participants were asked to complete a series of questionnaires and standardized assessments in-person or online (from home) within 3 days. Embedded in a survey about demographics and injury details was the following retrospective question: “Were you advised by at least one health professional to rest for more than 2 days after your injury?” Participants were considered to be exposed to guideline non-compliant prolonged rest advice when they answered this question affirmatively. Of 235 patients who met the eligibility criteria during the recruitment period, 150 agreed to participate, and of those 146 answered the rest advice exposure question, forming the sample for the present study.

### Outcome Measures

Participants reported their current productivity status by answering whether they had fully returned or partially returned to work/school, were on leave (e.g., sick or medical leave or short-term disability), or that the question did not apply to them (i.e., they were not working or going to school at the time of their injury).

The Rivermead Post-Concussion Symptom Questionnaire (RPQ) (27) is self-report inventory commonly used for mTBI research (28). It is a 16-item questionnaire that measures the severity of current symptoms (e.g., headache, fatigue, concentration difficulties) on a scale from 0 (“not experienced at all”) to 4 (“a severe problem”). A response of one indicates that a symptom is present but “no more of a problem” compared to pre-injury. Item scores of 2–4 are summed to create a total score ranging from 0 to 64.

The Patient Health Questionnaire (PHQ-9) (29, 30) is a brief, reliable, and valid depression screening measure. It queries the frequency of depression symptoms over the past 2 weeks. Items are summed to create a total score that can range from 0 to 27, where higher scores indicate worse depression symptoms.

The Generalized Anxiety Disorder (GAD-7) (31) screens for anxiety symptoms (e.g., feeling nervous, worrying too much, trouble relaxing) over the past 2 weeks. The total score ranges from 0 to 21, with higher scores reflecting worse anxiety.

### Statistical Analysis

Logistic regression was used to examine whether patient characteristics were associated with getting prolonged rest advice. No prior evidence was available to guide predictor selection. We hypothesized that health providers would be more likely to prescribe rest for more than 2 days (what they might view as conservative management) for patients they perceive (intentionally or inadvertently) to be vulnerable, such as women, older adults, and people with more severe injuries (indicated by the presence of loss of consciousness) or a history of prior mTBI(s) or psychiatric problems. Patients with access to financial compensation might be seen as more able to take time off work, so we also included this variable in the logistic regression model. Adjusted odds ratios were derived from the model in which all predictor variables were entered, and therefore reflect the *unique* association between the predictor and outcome, controlling for all other predictors.

We used generalized linear models to evaluate the relationship between advice to rest and clinical outcomes, including productivity status, post-concussion symptoms (RPQ), depression (PHQ-9), and anxiety (GAD-7). Data was missing for <5% of participants for all outcome variables except the RPQ. The total RPQ score was missing for 13% (*n* = 19) of participants. For participants who answered at least 13 of the 16 items (*n* = 10), missing item values were imputed with the participants' average item scores for the items they answered (rounded to a whole number). Participants who did not respond to three or more RPQ items (*n* = 9) and participants who responded “not applicable” to the productivity status question (*n* = 8) were excluded from the generalized linear models involving these outcome variables. We specified a multinomial probability distribution and cumulative logit link function for the ordinal productivity status outcome variable and a Gaussian probability distribution and identity link function for the continuous outcome variables.

Considering that participants who were exposed to prolonged rest advice might systematically differ from those who were not, we used regression-based propensity score adjustment to mitigate this potential bias. Specifically, we re-ran the generalized linear models with an additional covariate. The covariate was the predicted probability of group membership in the logistic regression model described above, where higher scores (closer to 1.0) represent greater likelihood of exposure to prolonged rest advice.

## Results

Participant characteristics are summarized in Table 1. The majority (82.9%) of participants were exposed to prolonged rest advice. As shown in Table 2, exposure to prolonged rest advice was not significantly associated with any of the hypothesized factors, including sex, age, loss of consciousness, or history of prior mTBIs or psychiatric problems.

**Table 1:** Demographic, injury, and initial assessment characteristics.

	**Full sample (*n* = 146)**	**Exposed to rest (*n* = 121)**	**Not exposed to rest (*n* = 25)**
**DEMOGRAPHICS**			
Age, M (SD)	40.6 (12.2)	39.8 (11.8)	44.4 (13.5)
Sex, n (%female)	98 (67.1%)	82 (67.8%)	16 (64%)
Ethnicity, *n* (%)			
Caucasian	88 (60.7%)	69 (57.5%)	19 (76%)
Other	57 (39.3%)	51 (42.1%)	6 (24%)
Education Level, *n* (%)			
High school or less	20 (13.7%)	16 (13.2%)	4 (16%)
Some college	24 (16.4%)	20 (16.5%)	4 (16%)
Technical degree/Diploma or associate degree	31 (21.2%)	26 (21.5%)	5 (20%)
Bachelor's degree	47 (32.2%)	38 (31.4%)	9 (36%)
Graduate/Professional degree	24 (16.4%)	21 (17.5%)	3 (12%)
**INJURY CHARACTERISTICS**			
Loss of Consciousness			
Yes	23 (15.8%)	18 (14.9%)	5 (22.7%)
No	99 (67.8%)	82 (67.8%)	17 (77.3%)
Unclear	7 (4.8%)	7 (5.8%)	0
Missing	17 (11.6%)	14 (11.6%)	0
Mechanism of Injury, *n* (%)			
Motor vehicle accident	68 (47.2%)	58 (48.3%)	10 (41.7%)
Fall	24 (16.7%)	21 (17.5%)	3 (12.5%)
Assault	2 (1.4%)	2 (1.7%)	0
Sports and recreation	24 (16.7%)	18 (15.0%)	6 (25%)
Other	26 (18.1%)	21 (17.5%)	5 (20.8%)
**HEALTH HISTORY**			
Previous mild traumatic brain injury, *n* (%)	55 (38.2%)	42 (35.3%)	13 (52%)
Pre-injury psychiatric problems, *n* (%)	54 (39.0%)	47 (38.8%)	10 (40%)
**INITIAL ASSESSMENT**			
Days to initial assessment, M (SD)	41.2 (26.7)	40.34 (26.8)	45.3 (26.6)
PHQ-9 total, M (SD)	13.3 (5.5)	13.6 (5.4)	12.3 (6.0)
GAD-7 total, M (SD)	9.8 (5.5)	9.9 (5.4)	9.2 (5.8)
RPQ total, M (SD)^*^	36.8 (13.6)	37.6 (13.6)	33.0 (13.8)
Access to Compensation, *n* (%)			
Yes	93 (63.7%)	78 (64.5%)	15 (60%)
No	39 (26.7%)	30 (24.8%)	9 (36%)
Unsure	14 (9.6%)	13 (10.7%)	1 (4%)
Return to Work/School Status, *n* (%)			
Full return	20 (13.3%)	14 (11.6%)	6 (24%)
Partial return	30 (20.5%)	24 (19.8%)	6 (24%)
On leave	88 (60.3%)	78 (64.5%)	10 (40%)
Not applicable	8 (5.5%)	5 (4.1%)	3 (12%)

* *Missing n = 9. PHQ-9, personal health questionnaire-9; GAD-7, generalized anxiety disorder-7; RPQ, rivermead post-concussion symptom questionnaire*.

**Table 2:** Logistic regression models.

**Variable**	**Odds ratio (95% CI)**		**Percentage advised to rest (>2 days)**
	**Unadjusted**	**Adjusted**	
Loss of consciousness	0.69 (0.23–2.10)	0.82 (0.25–2.73)	Present, 78.3%
			Absent, 84.0%
Sex	1.18 (0.48–2.91)	1.32 (0.47–3.69)	Female, 83.7%
			Male, 81.3%
History of mTBI(s)	0.50 (0.21–1.20)	0.50 (0.19–1.35)	one or more, 76.4%
			None, 86.5%
Pre-injury psychiatric problems	0.95 (0.40–2.30)	0.97 (0.35–2.69)	Present, 82.5%
			Absent, 83.1%
Access to compensation	1.21 (0.50–2.92)	1.41 (0.53–3.74)	Yes, 83.9%
			No, 81.1%
Age	0.97 (0.93–1.01)	0.97 (0.94–1.01)	–

Generalized linear modeling (see Table 3) revealed that participants who were exposed to prolonged rest advice had lower productivity at the time of assessment (2–10 weeks post-injury), B = −1.06, chi-squared(1) = 4.88, *p* = 0.027. This finding held after propensity score adjustment, B = −1.02, chi-squared(1) = 4.36, *p* = 0.037. The breakdown was: 64.5% in the exposure group vs. 40.0% in the control were on leave from work/school, 19.8 vs. 24% had partially returned to work/school, and 11.6 vs. 24% had fully returned to work/school. Exposure to prolonged rest advice was not a significant predictor of the post-concussion (RPQ), depression (PHQ-9), or anxiety (GAD-7) symptoms at the time of assessment (see Table 3), with trends favoring the non-exposed group on all variables (Table 1).

**Table 3:** Generalized linear models.

	**Single predictor model (exposure status only)**			**Two-predictor model (exposure status and propensity score)**		
	**B**	**Wald chi-square**	**Sig**.	**B**	**Wald chi-square**	**Sig**.
Productivity status	−1.055	4.875	0.027	−1.02	4.363	0.037
RPQ	−4.224	1.798	0.180	−5.091	2.111	0.146
PHQ-9	−1.102	0.746	0.388	−1.227	0.879	0.348
GAD-7	−0.647	0.248	0.619	−0.402	0.091	0.763

*PHQ-9, personal health questionnaire-9; GAD-7, generalized anxiety disorder-7; RPQ, rivermead post-concussion symptom questionnaire*.

## Discussion

Clinical practice guidelines for concussion and mTBI have moved away from “rest until asymptomatic” as the standard of care to promoting early, graded return to activity as tolerated after an initial 1–2 days of rest (19, 21). The present study found little evidence of progress with de-implementation of prolonged rest advice. The vast majority of patients with mTBI in our cohort (83%) reported being told by at least one health professional to rest for more than 2 days after their injury.

Understanding *who* is told to rest could inform knowledge translation efforts to de-implement this practice. If patients with certain characteristics are more likely to receive inappropriate advice, knowledge translation strategies could be tailored accordingly. We hypothesized that, for example, patients with worse injuries (i.e., had an acute loss of consciousness) and a history of prior mTBIs might be perceived to require more conservative management (longer rest time). We did not observe an association between prolonged rest advice and any measured demographic, health history, or injury variable.

Another aim of the present study was to check for empirical evidence consistent with the hypothesis that prolonged rest advice is associated with delayed return to productivity and elevated risk of depression (17). Prescribed rest is likely ineffective, that is, probably does not facilitate recovery (11, 13, 14), but it is unclear whether this clinical intervention has adverse effects. Patients who are (inappropriately) told that prolonged rest is therapeutic may be more cautious in resuming their usual activities. In turn, isolation from socialization and valued activities may precipitate depression (32). We compared the clinical outcomes of patients who reported being advised to rest for a prolonged period (>2 days after their injury) to patients who denied receiving such (guideline non-compliant) advice. Our main finding was that the two groups looked similar (at mean = 41.2 days post-injury) with respect to post-concussion symptoms, anxiety, and depression; however, participants who were exposed to prolonged rest advice were significantly less likely to have returned to their pre-injury work or school status.

The present study has important limitations. It provides a snapshot of de-implementation at one point in time (2017–2018), in one geographic region. It also does not provide any granularity with respect to who delivered the rest advice (which health professionals), where the communication occurred (e.g., in an Emergency Department vs. a primary care office), or the exact content of the communication. Our methodology relied on the patient's recall of what they were told, which may be biased by misunderstanding or misremembering, but is arguably most relevant to their clinical outcomes. Although participants in the exposure group were told to rest for more than 2 days by “at least one” health professional, it is entirely possible that they were given contradictory advice by other health professionals and/or were exposed to contradictory advice from non-health professionals and written or web-based information sources. Considering this fact, it is somewhat surprising that we detected a significant association between exposure status and productivity outcomes. It is important to emphasis that this was an observational study, not a randomized trial. It may not have been random who was exposed to rest vs. not. No measured patient factors were statistically associated with exposure, which means either that (i) the groups were well-matched, approximating a randomized trial, or that (ii) important confounds went unmeasured. That is, our attempt to control for potential confounds with regression-based propensity matching may have been unnecessary or ineffective. Generalizability of the study findings may be limited by selection bias. Participants were recruited from an outpatient specialty clinic setting and therefore our sample did not include patients who recovered well without seeking care. To the extent prolonged rest advice is associated slow recovery, we may have over-sampled patients with exposure to such advice. Further contributing to selection bias, almost 40% of eligible patients did not enroll in the study.

In summary, we found that prolonged rest advice continues to be dispensed widely and without apparent consideration of individual patient or injury factors. We also found that patients who received prolonged rest advice were less likely to have completely returned to work or school at ~6 weeks post-injury. De-implementation efforts are warranted, and could be facilitated by research into health provider and environmental factors that influence the practice of advice to rest after mTBI.

## Ethics Statement

This study was carried out in accordance with the recommendations of name of guidelines, name of committee; with written informed consent from all subjects. All subjects gave written informed consent in accordance with the Declaration of Helsinki. The protocol was approved by the University of British Columbia Clinical Research Ethics Board.

## Author Contributions

NS obtained funding, conceived of the study, oversaw data collection, and co-wrote the first draft of the manuscript. TO refined the research questions, carried out the statistical analyses, and co-wrote the first draft of the manuscript. Both authors reviewed and approved the final version.

### Conflict of Interest Statement

NS receives research salary support from a Michael Smith Foundation for Health Research Health Professional Investigator Award. He has also received fees for private neuropsychology consulting. He is a member of Highmark Interactive's Medical Advisory Board (<5% full time equivalency). The remaining author declares that the research was conducted in the absence of any commercial or financial relationships that could be construed as a potential conflict of interest.
